# Supplementary material for: Sustained-input switches for transcription factors and microRNAs are central building blocks of eukaryotic gene circuits
Source: Genome Biol. 2013 Aug 23;14(8):R85. doi: 10.1186/gb-2013-14-8-r85 (PMC4054853; doi:10.1186/gb-2013-14-8-r85)
Supplement: Additional file 5 — HTML Browsable Motif Output. Zipped folder containing all WaRSwap and FANMOD motif output, viewable in a web browser. [file gb-2013-14-8-r85-S5.ZIP › HTML_browsable_motif_output/FANMOD_ath_tair9/sigs_fanmodm-2000.pvals.heatmaps.html/motif_id_38_011011002_tftype_ath_upstream_-2000_0.html]

```
BG_MODEL = FANMOD
MOTIF_ID = 38_011011002
TF_TYPE = ath
UPSTREAM = -2000_0


PVals
FN_0.2	FN_0.4	FN_0.6	FN_0.8
dg_60.genes	0	0	0.479	0
dg_70.genes	0	0	0.007	0.001
dg_80.genes	0	0	0.071	0.147

ZScores
FN_0.2	FN_0.4	FN_0.6	FN_0.8
dg_60.genes	4.097	5.156	0.022	3.716
dg_70.genes	3.451	5.663	2.429	2.957
dg_80.genes	5.37	5.545	1.46	1.021

StDevs
FN_0.2	FN_0.4	FN_0.6	FN_0.8
dg_60.genes	107.416	92.738	47.061	18.143
dg_70.genes	82.239	71.508	36.608	14.655
dg_80.genes	53.067	49.782	26.918	11.062
```
